# Fractal-driven self-supervised learning enhances early-stage lung cancer GTV segmentation: a novel transfer learning framework

**DOI:** 10.1007/s11604-025-01865-8

**Published:** 2025-09-15

**Authors:** Ryota Tozuka, Noriyuki Kadoya, Arata Yasunaga, Masahide Saito, Takafumi Komiyama, Hikaru Nemoto, Hidetoshi Ando, Hiroshi Onishi, Keiichi Jingu

**Affiliations:** 1 Department of Radiation Oncology, Tohoku University Graduate School of Medicine, Sendai, Japan; 2 Department of Radiology, University of Yamanashi, Yamanashi, Japan; 3 Department of Computer Science, University of Yamanashi, Yamanashi, Japan

**Keywords:** Radiotherapy, Segmentation, Lung, AI, Transfer learning

## Abstract

**Purpose:**

To develop and evaluate a novel deep learning strategy for automated early-stage lung cancer gross tumor volume (GTV) segmentation, utilizing pre-training with mathematically generated non-natural fractal images.

**Materials and methods:**

This retrospective study included 104 patients (36–91 years old; 81 males; 23 females) with peripheral early-stage non-small cell lung cancer who underwent radiotherapy at our institution from December 2017 to March 2025. First, we utilized encoders from a Convolutional Neural Network and a Vision Transformer (ViT), pre-trained with four learning strategies: from scratch, ImageNet-1K (1,000 classes of natural images), FractalDB-1K (1,000 classes of fractal images), and FractalDB-10K (10,000 classes of fractal images), with the latter three utilizing publicly available models. Second, the models were fine-tuned using CT images and physician-created contour data. Model accuracy was then evaluated using the volumetric Dice Similarity Coefficient (vDSC), surface Dice Similarity Coefficient (sDSC), and 95th percentile Hausdorff Distance (HD95) between the predicted and ground truth GTV contours, averaged across the fourfold cross-validation. Additionally, the segmentation accuracy was compared between simple and complex groups, categorized by the surface-to-volume ratio, to assess the impact of GTV shape complexity.

**Results:**

Pre-trained with FractalDB-10K yielded the best segmentation accuracy across all metrics. For the ViT model, the vDSC, sDSC, and HD95 results were 0.800 ± 0.079, 0.732 ± 0.152, and 2.04 ± 1.59 mm for FractalDB-10K; 0.779 ± 0.093, 0.688 ± 0.156, and 2.72 ± 3.12 mm for FractalDB-1K; 0.764 ± 0.102, 0.660 ± 0.156, and 3.03 ± 3.47 mm for ImageNet-1K, respectively. In conditions FractalDB-1K and ImageNet-1K, there was no significant difference in the simple group, whereas the complex group showed a significantly higher vDSC (0.743 ± 0.095 vs 0.714 ± 0.104, *p* = 0.006).

**Conclusion:**

Pre-training with fractal structures achieved comparable or superior accuracy to ImageNet pre-training for early-stage lung cancer GTV auto-segmentation.

## Introduction

Defining the target area for radiation therapy, specifically segmenting the Gross Tumor Volume (GTV), is crucial in treatment planning. However, this manual segmentation by radiation oncologists is labor-intensive and suffers from significant inter-observer variability, which compromises the quality and reproducibility of treatment plans [1–3]. To mitigate these challenges, deep learning-based automated segmentation methods have emerged as a promising solution [3].

Previous studies trained deep learning models with extensive patient and multi-modality data (e.g., PET/CT) [4–8]. However, such data collection and training are time-consuming, especially for smaller institutions. Furthermore, even high-quality data-trained models often face inference noise due to varying equipment, CT acquisition methods, and quality control across institutions, hindering universal applicability [9]. Thus, transfer learning, where a model is pre-trained on readily available non-medical images and then fine-tuned with institution-specific medical data, is effective [10, 11].

To maximize fine-tuning effectiveness with limited data, a robust large-scale pre-training dataset is essential. ImageNet, a representative natural image dataset, has shown utility even in medical image segmentation [12]. Yet, many natural image datasets, including ImageNet, contain data vastly different from medical conditions like lung cancer lesions (e.g., cats, birds). This discrepancy can cause *Negative Transfer* during transfer learning, which in turn reduces accuracy [13–15]. These datasets also have human-labeled errors and biases, and unclear licensing, posing copyright issues [16, 17].

**Fig. 1:**
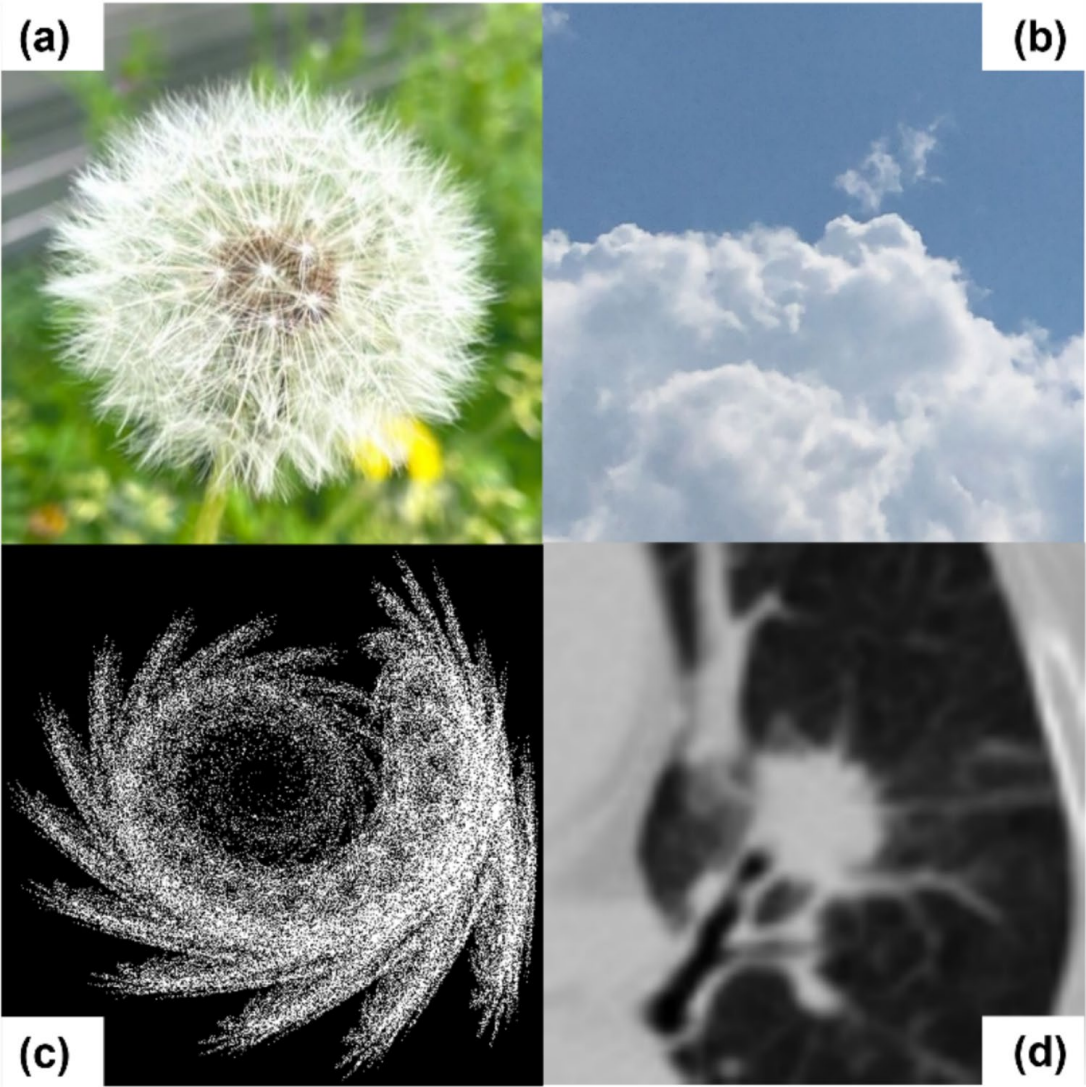
Examples of fractal structures. **a** Dandelion seed head, **b** cloud, **c** mathematically generated synthetic fractal image, and **d** lung cancer tumor, all of which exhibit fractal properties

To resolve these problems, we focused on fractal structures. Fractal structures are ubiquitous geometrical patterns in nature, such as plants (Fig. 1a) and clouds (Fig. 1b). Kataoka et al. showed that pre-training with mathematically generated fractal structures (Fig. 1c) achieved ImageNet-comparable classification accuracy [18]. We propose using these fractal images for pre-training via self-supervised learning (SSL) in a lung cancer GTV auto-segmentation model. Crucially, fractal structures are common in nature, and lung cancer tumors in CT images exhibit fractal properties (Fig. 1d) [19]. This implies that pre-training with images that intrinsically contain tumor-like shapes, rather than images vastly different from tumor morphology like those in ImageNet, may enable more effective transfer learning. Furthermore, by adopting SSL, where the parameters used to generate the fractal images serve as the ground truth labels, additional segmentation of pre-training data was not required. Moreover, since fractal images are artificial images generated by mathematical equations, they are free of licensing issues, which facilitates the public sharing and availability of the data and pre-trained models [18, 20].

This study aims to develop a lung cancer GTV auto-segmentation model based on publicly available models pre-trained with fractal images, and to compare its utility with existing methods.

## Materials and methods

### Patients and equipment

In this retrospective study, we consecutively enrolled 104 patients (36–91 years old; 81 males; 23 females; GTV volume range: 1.361–164.1 cc; mean GTV volume: 17.01 cc) with peripheral early-stage non-small cell lung cancer who underwent radiotherapy at our institution from December 2017 to March 2025. This study was approved by the Institutional Ethics Committee of the University of Yamanashi (Approval No.: CS0010). The requirement for individual informed consent was waived because this was a retrospective study. Information about the study was disclosed on our institution's website, providing patients with the opportunity to opt out. To ensure patient privacy, the data were de-identified and assigned a unique study-specific number.

Planning CT images were acquired using an Aquilion LB (Canon Medical Systems, Japan) with a matrix size of 512 × 512, slice thickness of 2.0 mm, and pixel size of 1.074 × 1.074 mm. All planning CT images were obtained during inspiratory breath-hold using Abches (APEX Medical Inc., Japan). GTV contours were delineated by a radiation oncologist with over 7 years of experience using the commercial treatment planning system Pinnacle (Philips Radiation Oncology Systems, USA). Subsequently, all cases were reviewed and approved by at least three senior radiation oncologists (with over 10 years of experience) during departmental conferences.

All image processing, computation, and statistical analyses in this study were performed using Python (version 3.12). The segmentation models were implemented using the PyTorch framework (version 2.6.0).

### Data preprocessing

In GTV auto-segmentation tasks, the tumor size is typically very small relative to the entire CT image, which leads to a severe class imbalance between the tumor and the background [4, 5]. Additionally, ResNet-18, which we adopted as the encoder model in this study, repeatedly halves the image size through downsampling. Therefore, setting the pixel size to a power of two allows for stable computation, as it prevents remainders and the need for exception handling. For these reasons, all CT images and corresponding GTV binary masks were cropped to a 64 × 64 pixels (68.736 × 68.736 mm) region of interest (ROI) centered on the GTV centroid, as this was the smallest power-of-two size that could fully encompass the tumor while minimizing the inclusion of unnecessary background. All GTVs in the cases included in this study were fully contained within this ROI, with axial maximum diameters ranging from 12.9 to 65.6 mm (mean: 27.2 mm).

### Fractal image generation and FractalDB construction

**Fig. 2:**
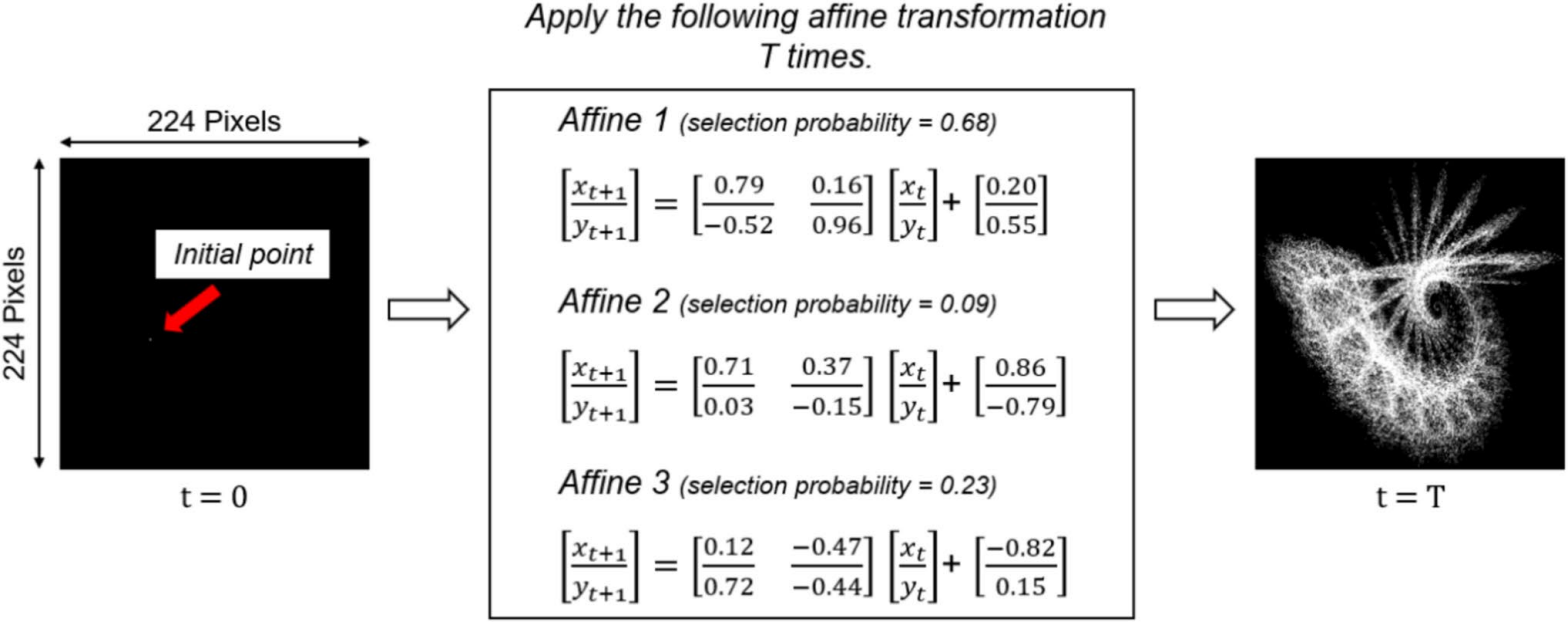
Workflow of fractal image generation. A fractal image is generated by starting from an initial point at *t* = 0 and repeatedly applying randomly selected affine transformations until *t* = *T*

The fractal images used in this study are mathematically generated synthetic images that mimic ubiquitous fractal structures found in nature. The dataset composed of these images is named FractalDB [18]. The procedure for generating fractal images is shown in Fig. 2. Although the fractal image generation algorithm has been detailed by Kataoka et al., we provide a brief overview below, as it is a core technology of this study.

Iterated Function Systems (IFS) were used for image generation. An IFS is a family of functions composed of $N$ affine transformations, defined in the following form:

$$\text{IFS}= \left\{X;{w}_{1}, {w}_{2}, \dots, {w}_{N}; {p}_{1}, {p}_{2}, \dots, {p}_{N}\right\}$$

Here, $X$ is a complete metric space, ${w}_{i}$ are affine transformations, ${p}_{i}$ is the probability that ${w}_{i}$ is selected. Each affine transformation ${w}_{i}$ is defined in the following form, where ${\theta }_{i}=({a}_{i}, {b}_{i}, {c}_{i}, {d}_{i}, {e}_{i}, {f}_{i})$ represents the affine transformation parameters:

$${w}_{i}\left({{\varvec{x}}}_{{\varvec{t}}+1};{\theta }_{i}\right)=\left[\begin{array}{cc}{a}_{i}& {b}_{i}\\ {c}_{i}& {d}_{i}\end{array}\right]{{\varvec{x}}}_{{\varvec{t}}}+\left[\begin{array}{c}{e}_{i}\\ {f}_{i}\end{array}\right]$$

$${\varvec{x}}=\left[\begin{array}{c}x\\ y\end{array}\right]$$

By iteratively applying these transformations any number of times, it's possible to generate self-similar fractal patterns. For image generation, many points are rendered to form a 2D fractal image by starting from an initial point $({x}_{0}, {y}_{0})$ and repeatedly calculating the selected transformation ${w}_{i}$ based on its probability ${p}_{i}$.

At this point, when the IFS configuration parameters (the number of affine transformations $N$, the affine transformation parameters ${\theta }_{i}$, and the probabilities of selecting each affine transformation ${p}_{i}$) are randomly determined, the random parameter set $\Theta$ can be expressed as follows:

$$\Theta ={\left\{({\theta }_{i}, {p}_{i})\right\}}_{i=1}^{N}$$

Kataoka, et al. define this as a fractal category. Furthermore, by slightly varying these parameters, we can generate image variations (referred to as instances) within a fractal category.

FractalDB is a fractal image dataset composed of these fractal categories and the instances contained within them. In other words, a fractal category in FractalDB is analogous to a class in ImageNet, and the images within each class correspond to instances. Notably, while the fractal category is defined by the IFS configuration parameters $\Theta$, FractalDB trains the model to estimate these parameters as a task. This approach allows for the automatic assignment of pseudo-labels to fractal images, even without explicit ground truth labels like cats or birds found in ImageNet.

This study utilized publicly available models pre-trained on the FractalDB dataset, which was constructed by Kataoka et al. According to the original publication, the parameters used to generate FractalDB were configured as follows: the IFS parameters $a$ through $f$, as well as $p$, were set within the range of − 1.0 to 1.0, with the constraint that the sum of the $p$ values equals 1. The number of applied affine transformations was 200,000 per image. Additionally, to increase the variation in fractal shapes, each parameter set was multiplied by a weight ranging from 0.8 to 1.2, in increments of 0.1 [18].

### Pre-trained model

We evaluated DeepLabV3+, a convolutional neural network (CNN)-based encoder-decoder model, and Dense Prediction Transformers (DPT), a Transformer-based encoder-decoder model, for 2D lung cancer GTV auto-contouring. The ResNet-101 backbone of DeepLabV3+ was replaced with a more lightweight ResNet-18, as its deep structure would excessively downsample the small 64 × 64 inputs, resulting in feature maps with insufficient spatial resolution [21]. DPT, originally using ViT-Hybrid or ViT-Large, was adapted using the more efficient Data-efficient image Transformers Tiny (DeiT-Tiny) backbone to avoid overfitting with our limited cases [22]. These models were built using the segmentation_models_pytorch (v0.5.0) and pytorch-image-models libraries (v1.0.15).

We investigated four distinct training strategies for the encoders (i.e., ResNet-18 or DeiT-Tiny) of both models: training from scratch (random initialization), pre-trained on ImageNet-1K (ImageNet-1K_PT), pre-trained on FractalDB-1K (FractalDB-1K_PT), and pre-trained on FractalDB-10K (FractalDB-10K_PT). Due to computational resource constraints, we used publicly available pre-trained models for these three strategies. For the ImageNet-1K_PT model, we loaded the standard weights provided by the segmentation_models_pytorch library. For the FractalDB models, we used the versions publicly released by Kataoka et al. (https://github.com/hirokatsukataoka16/FractalDB-Pretrained-ResNet-PyTorch) and Nakashima et al. (https://github.com/nakashima-kodai/FractalDB-Pretrained-ViT-PyTorch).

Training from scratch and ImageNet-1K_PT are commonly used approaches in semantic segmentation tasks and serve as the baseline comparisons in this study. FractalDB-1K, containing 1 million images across an equivalent number of classes (1,000) to ImageNet-1K (which has 1.28 million images), was tested to evaluate if comparable or exceeding accuracy could be achieved. Additionally, we employed FractalDB-10K to leverage the inherent scalability of the fractal dataset. This dataset, containing 10 million images across 10 times as many classes (10,000) as FractalDB-1K, was tested to determine if superior accuracy could be achieved.

Unlike ImageNet, which contains ground-truth labels (e.g., cats, birds) for training models on a classification task, FractalDB lacks such annotations. To tackle this, FractalDB therefore formulates a pretext task where the model is trained to estimate the fractal categories$\Uptheta$ (introduced in the "Fractal image generation and FractalDB construction section"), using them as pseudo-labels. This approach, known as SSL, offers the considerable advantages of reducing manual labeling effort and mitigating potential human-induced biases [23, 24].

### Transfer learning

The encoders used in this study, ResNet-18 and DeiT-Tiny, were pre-trained on image classification tasks using the ImageNet or FractalDB datasets. To adapt these models for lung cancer segmentation, architectural modifications are necessary. Specifically, the layers specialized for the classification task need to be removed, and a segmentation decoder needs to be connected.

When ResNet-18 is used as the backbone for DeepLabV3+, its final fully-connected layer, which serves as the classification head, is removed. The high-level feature maps from the encoder are fed into the Atrous Spatial Pyramid Pooling module of DeepLabV3+, while the spatially rich, low-level feature maps are fused with the decoder during the upsampling process [21].

For DeiT-Tiny, used as the backbone for the DPT, the classification head and the CLS token are discarded. The remaining patch tokens output by the encoder are transformed into multi-resolution 2D feature maps by DPT's Reassemble module. These feature maps are then progressively fused and upsampled by a subsequent convolutional decoder to generate the final segmentation mask [22].

Constructing these model architectures from scratch is a considerably complex and time-consuming process. To streamline development, this study leveraged the PyTorch deep learning framework, which supports these intricate operations. Specifically, high-level libraries such as segmentation-models-pytorch enable the rapid construction of transfer learning models simply by specifying the desired encoder and segmentation architecture. All models in this study were implemented using this high-level library, built upon the PyTorch framework.

### Fine-tuning process

**Fig. 3:**
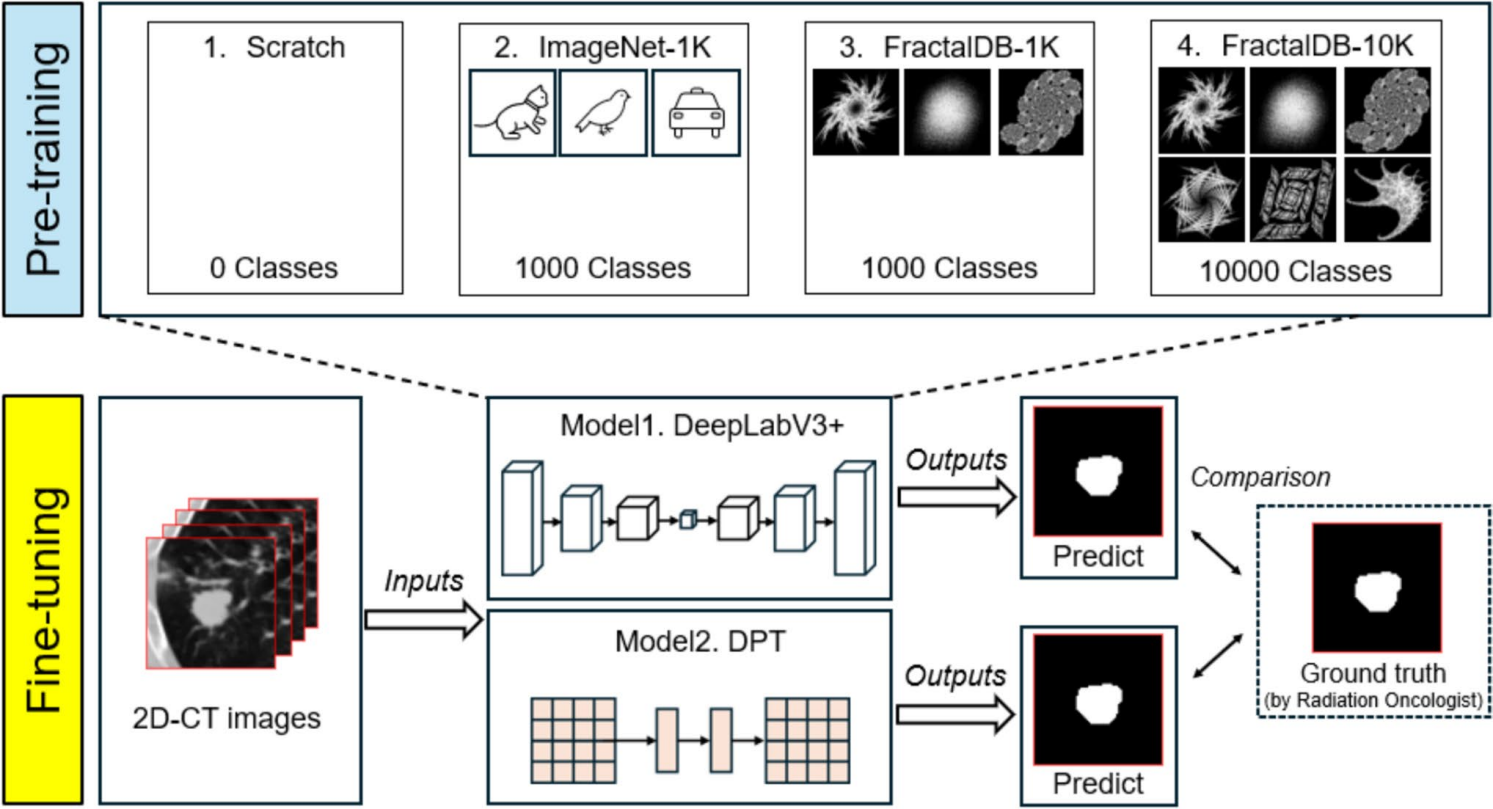
Schematic overview of the entire workflow in this study

Figure 3 illustrates an overview of this study. We performed fine-tuning of the models using a dataset of 104 patient cases, consisting of 2D CT images and their corresponding 2D GTV binary masks. Of these, 78 cases were allocated to the training dataset (63 for training and 15 for validation), and 26 cases were assigned to the test dataset. We therefore used the validation set not for hyperparameter tuning, but for selecting the optimal training epoch, specifically the one that yielded the lowest validation loss. Given the limited number of training samples, we employed fourfold cross-validation to appropriately evaluate the models' generalization performance (the detailed breakdown of the number of 2D slices for cross-validation is provided in Table 1). To prevent overfitting to the training dataset, we employed on-the-fly data augmentation using the Albumentations library. The specific set of transformations, detailed in Table 2, was based on the methods described in the official tutorials of segmentation-models-pytorch (https://github.com/qubvel-org/segmentation_models.pytorch) and the original Albumentations paper [25].

**Table 1:** Data distribution for the fourfold cross-validation

Number of 2D slices	Fold 1	Fold 2	Fold 3	Fold 4
Training dataset	787	809	815	761
Validation dataset	204	196	207	205
Test dataset	337	323	306	362

**Table 2:** Data augmentation parameters and settings

Transformation	Parameters	Probability (%)
HorizonalFlip	–	50
ShiftScaleRotate	scale_limit = 0.5, rotate_limit = 0, shirft_limit = 0.1	100
GaussNoise	–	20
Perspective	–	50
OneOf(CLAHE, RandomBrightnessContrast, RandomGamma)	–	90
OneOf(Sharpen, Blur, MotionBlur)	blur_limit = 3	90
OneOf(RandomBrightnessContrast, HueSaturationValue)	–	90

We created a total of eight distinct deep learning (DL) models by combining the two base models with four different pre-training strategies. While DeepLabV3+ directly processed the 64 × 64 ROIs, DPT required the input to be resized to 224 × 224 to match its DeiT-Tiny encoder. For the DPT model's training, both CT and mask images were upsampled to 224 × 224 using nearest-neighbor interpolation. The resulting predicted masks were then downsampled back to the original 64 × 64 resolution using the same method for evaluation. For all DL models, we fine-tuned the entire model using the Adam optimizer with a batch size of 8 for up to 50 epochs. To prevent the disruption of pre-trained weights, we employed a learning rate warm-up strategy; the rate started at 2e-5, for the first epoch, was gradually increased to the target rate of 1e-4 by the fifth epoch, and then remained constant. This overall approach was chosen because it is known that aggressive fine-tuning can degrade performance by disrupting the learned pre-trained parameters [26].

### Quantitative evaluation of segmentation

We compared the GTV segmentations predicted by our models with manually delineated contours by radiation oncologists. Given the strengths and weaknesses of various contour similarity metrics, we utilized a combination of several for evaluation in this study [27].

The volume dice coefficient ($\text{vDSC}$) quantifies volumetric overlap between two volumes, A and B, defined as:

$$\text{vDSC}=\frac{2\left|A\cap B\right|}{\left|A\right|+\left|B\right|}$$

The $\text{vDSC}$ is a very simple metric: 1 for complete overlap and 0 for no overlap. Despite its simplicity, it has drawbacks, including weak correlation with clinical validity and overestimation for larger volumes. Nevertheless, we adopted $\text{vDSC}$ as it's a widely used and frequently reported metric for quantifying region similarity [27].

The surface dice coefficient ($\text{sDSC}$), a newer metric proposed by Nikolov et al., addresses $\text{vDSC}$ shortcomings by evaluating contour agreement, not volume [28]. For two contours, we extract their surface point clouds, defined as ${S}_{A}$ and ${S}_{B}$ from their respective 3D volumes. Furthermore, an allowed tolerance $\tau (\text{mm})$ is introduced, and the point clouds that match within $\tau$ are defined as ${S}_{A}^{\tau }$ and ${S}_{B}^{\tau }$, respectively. The $\text{sDSC}$ is then defined as:

$$\text{sDSC}\left(\tau \right)=\frac{\left|{S}_{A}^{\tau }\right|+\left|{S}_{B}^{\tau }\right|}{\left|{S}_{A}\right|+\left|{S}_{B}\right|}$$

As $\text{sDSC}$ focuses on the contours, it's particularly useful in tasks like radiation therapy where peripheral accuracy is crucial. In this study, we used a commonly accepted tolerance value of $\tau =1 \text{mm}$ [29, 30].

Hausdorff Distance ($\text{HD}$) measures the maximum of the shortest distances from any point in one contour to the other contour. $\text{HD}$ is known to have a low correlation with the $\text{vDSC}$ and can thus complement its shortcomings [31]. However, its sensitivity to outliers, due to relying on a maximum value, led us to adopt the 95th percentile Hausdorff Distance ($\text{HD}95$), which is commonly used to mitigate this drawback [27].

To evaluate the differences in segmentation accuracy among pre-training strategies, we used the manually delineated contours by radiation oncologists as ground truth and assessed them using $\text{vDSC}$, $\text{sDSC}$, and $\text{HD}95$. For statistical comparison among groups, we employed the Friedman test, which is suitable for non-parametric repeated measures data. If a significant difference was observed with the Friedman test, we performed pairwise comparisons using the Wilcoxon signed-rank test to clarify differences between individual groups, with Holm's method applied for multiple comparison correction. Statistical analysis was conducted using Python (scipy and statsmodels libraries). A significance level of less than 5% (*p* < 0.05) was set.

### Subgroup analysis

Our hypothesis posits that pre-training with fractal images will enable models to more accurately segment complex shapes exhibiting fractal structures, such as lung cancer tumors. Specifically, we anticipate that the FractalDB-1K_PT will be more capable of handling complex shapes compared to the ImageNet-1K_PT.

To demonstrate this hypothesis, we conducted a subgroup analysis focusing on tumor shape complexity to compare the segmentation accuracy changes of each model. We divided the 104 cases used in this study into two groups based on their shape complexity index: the Lower 52 cases (simple group) and the upper 52 cases (complex group). We used the surface-to-volume ratio ($\text{SVR}$) as the complexity metric. $\text{SVR}$, calculated by dividing a GTV’s surface area by its volume, is higher for intricate, non-spherical shapes and Lower for simple, sphere-like shapes. Consequently, the $\text{SVR}$ for the simple group was 0.325 ± 0.058, and for the complex group, it was 0.502 ± 0.073. For this comparison, we did not retrain the models on each group; instead, we used the inference results from the fourfold cross-validation performed on all 104 cases as described in the "Fine-tuning process" section.

## Results

### Overall results

**Fig. 4:**
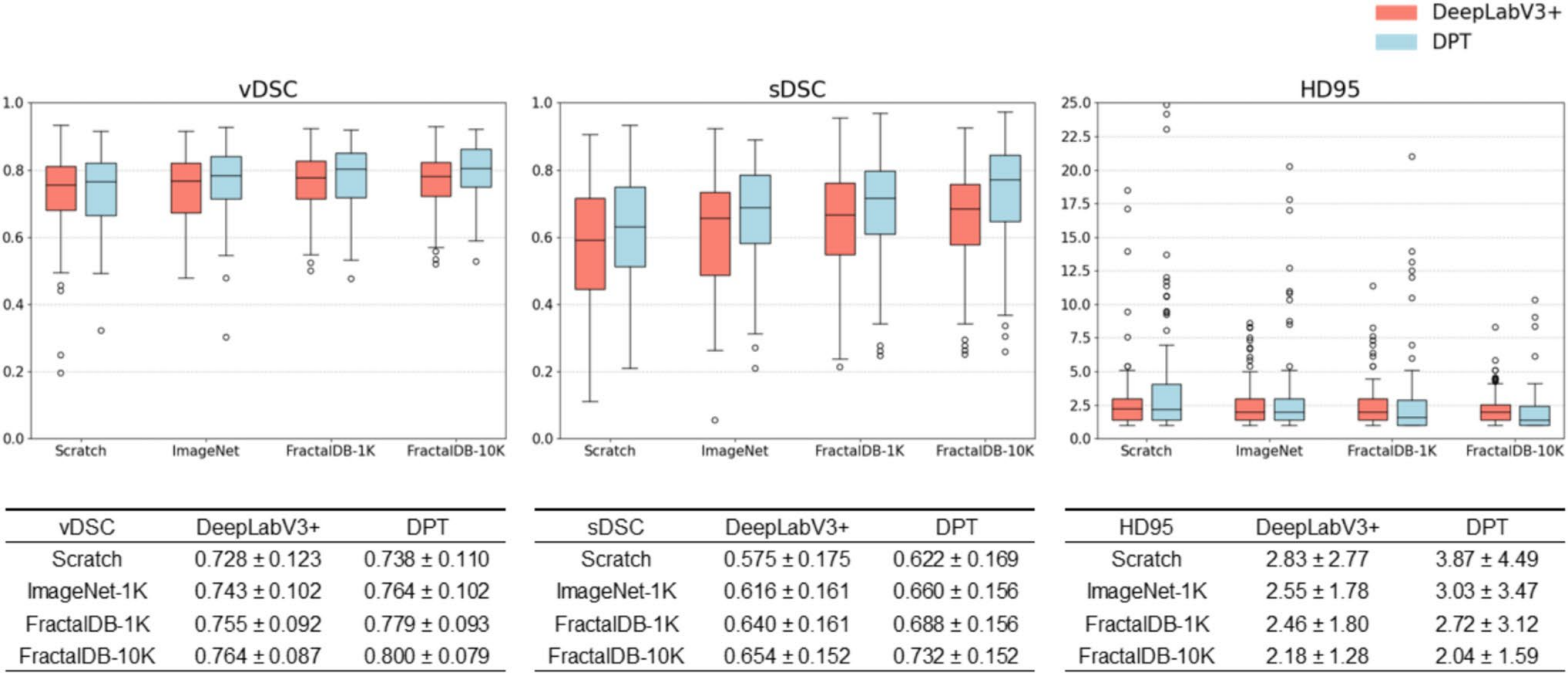
Segmentation accuracy of the DeepLabV3+ and DPT. The error bars represent the 95% confidence interval, the horizontal line inside the box indicates the median, and the black circles represent outliers. In both models, FractalDB-10K_PT achieved the best mean accuracy across all metrics, followed by FractalDB-1K_PT, ImageNet-1K_PT, and scratch in decreasing order of accuracy. $\text{vDSC}$, volume dice coefficient; $\text{sDSC}$, surface dice coefficient; $\text{HD}95$, 95th percentile Hausdorff Distance

Segmentation accuracy for DeepLabV3+ and DPT models, trained from scratch, pre-trained with ImageNet-1K_PT, FractalDB-1K_PT, and FractalDB-10K_PT, respectively, are presented in Fig. 4. In all metrics, FractalDB-10K_PT yielded the highest prediction accuracy, followed by FractalDB-1K_PT, ImageNet-1K_PT, and scratch in descending order of accuracy.

When comparing accuracy between the two models, DPT demonstrated higher $\text{vDSC}$ and $\text{sDSC}$ for all pre-training strategies (e.g., for FractalDB-1K: $\text{vDSC}$ of 0.755 ± 0.092 for DeepLabV3+ vs. 0.779 ± 0.093 for DPT; $\text{sDSC}$ of 0.640 ± 0.161 for DeepLabV3+ vs. 0.688 ± 0.156 for DPT). However, DPT showed reduced accuracy in $\text{HD95}$ for scratch, ImageNet-1K_PT, and FractalDB-1K_PT (e.g., for FractalDB-1K: 2.46 ± 1.80 mm for DeepLabV3+ vs. 2.72 ± 3.12 mm for DPT).

**Fig. 5:**
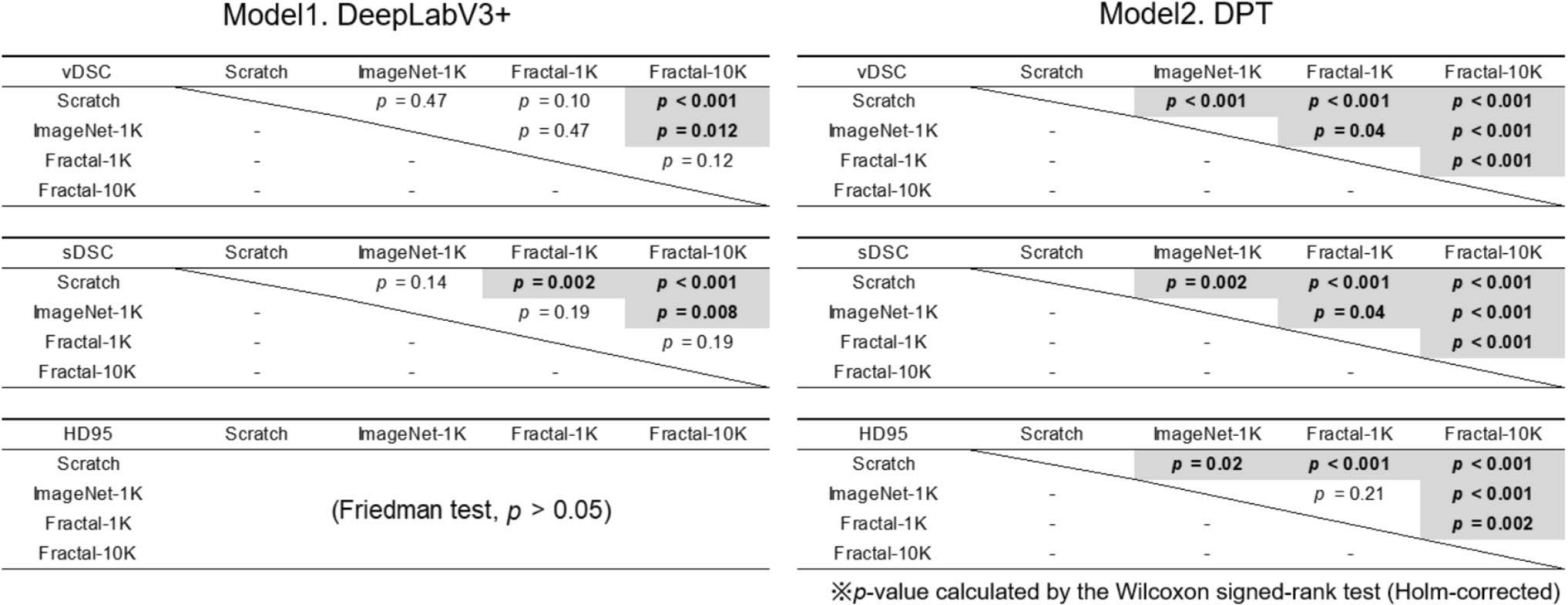
Statistical analysis of accuracy metrics across different pre-training strategies for both models. Numbers in bold text with a gray background indicate combinations with a significant difference (*p* < 0.05) in the Wilcoxon signed-rank test (Holm-corrected), following the confirmation of a significant difference by the Friedman test. $\text{vDSC}$, volume dice coefficient; $\text{sDSC}$, surface dice coefficient; $\text{HD}95$, 95th percentile Hausdorff Distance

Statistical analysis was conducted to evaluate the differences in accuracy among the four pre-training strategies. The results of the statistical analysis for each model are presented in Fig. 5. For the DeepLabV3+, significant differences in $\text{vDSC}$ and $\text{sDSC}$ were observed between some pre-training strategies. For the DPT, significant differences were found in all comparisons except for $\text{HD95}$ between ImageNet-1K_PT and FractalDB-1K_PT.

### Subgroup analysis results

**Fig. 6:**
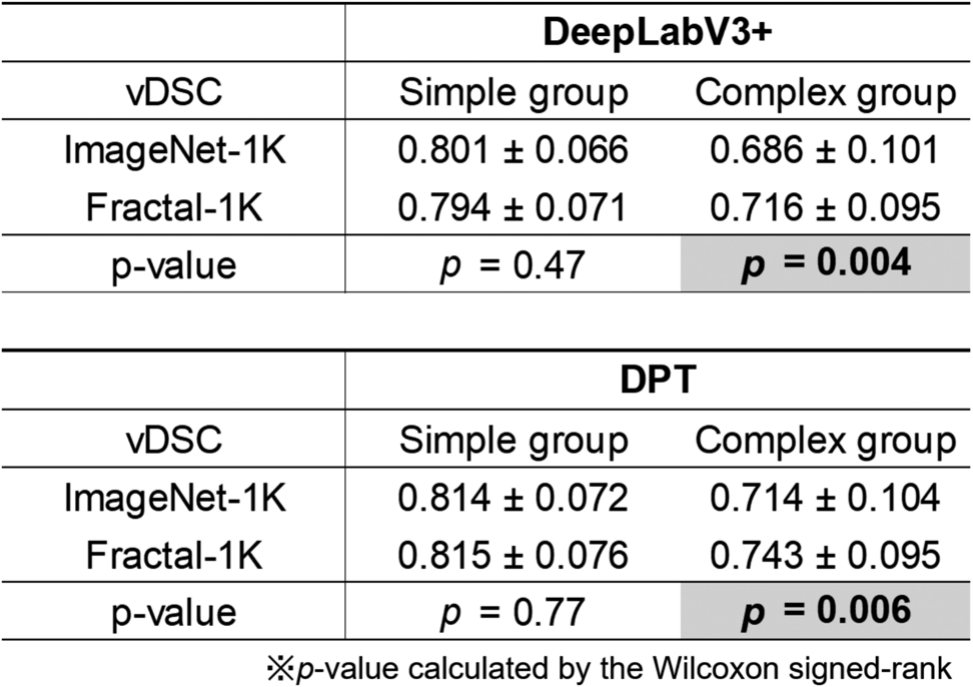
Comparison of $\text{vDSC}$ between the two models on Simple and Complex groups. Numbers in bold text with a gray background indicate combinations with a significant difference (*p* < 0.05) in the Wilcoxon signed-rank test. $\text{vDSC}$, volume dice coefficient

**Fig. 7:**
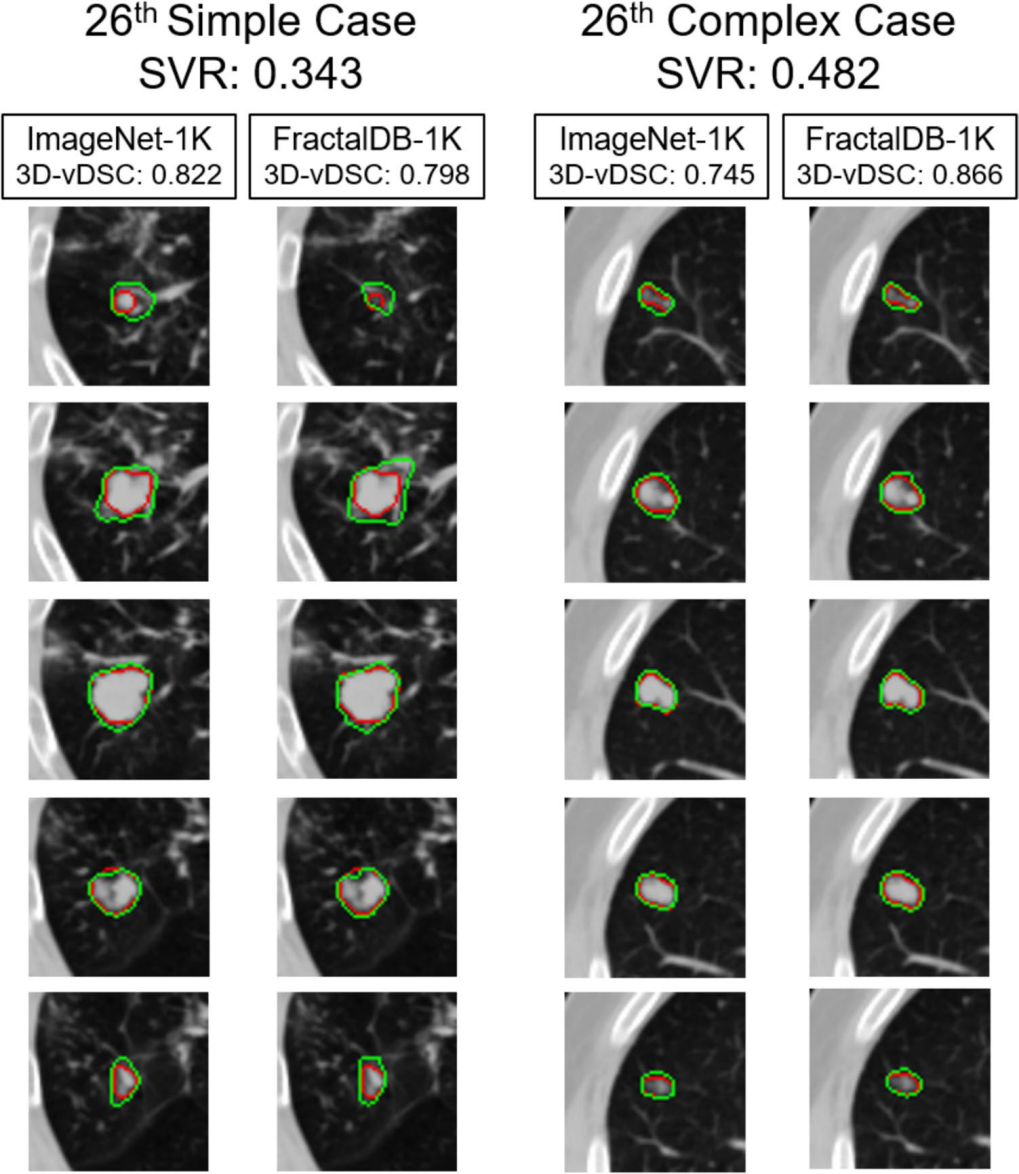
Typical examples of simple and complex cases. These images represent the median cases (i.e., the 26th simplest/most complex) when the 104 patient cases were divided into simple and complex groups based on their $\text{SVR}$. The red line indicates the ground truth contour defined by radiation oncologists, while the green line represents the segmentation contour predicted by the model (DPT). $\text{SVR}$, surface-to-volume ratio; $\text{vDSC}$, volume dice coefficient

Figure 6 presents the prediction accuracy ($\text{vDSC}$) and statistical analysis of ImageNet-1K_PT and Fractal-1K_PT models when the 104 cases were divided into simple and complex groups using the $\text{SVR}$. Representative cases for both the simple and complex groups (specifically, the 26th simplest and 26th most complex cases out of 52 in each group) are shown in Fig. 7.

In the simple group, no significant differences in accuracy were observed among the pre-training strategies, regardless of the model type. Conversely, in the complex group, FractalDB-1K_PT yielded significantly higher $\text{vDSC}$ compared to ImageNet-1K_PT, suggesting its superior suitability for segmenting complex shapes.

## Discussions

Previous reports show that lung cancer lesions on CT images exhibit fractal properties [19]. Applying this, we hypothesized that pre-trained DL models with fractal structures would achieve more accurate GTV auto-segmentation than existing methods like pre-training from scratch or ImageNet. Our results confirmed that pre-training with fractal structures improved segmentation accuracy when compared to other pre-training approaches. To our knowledge, no prior research has applied fractal structures to lung cancer segmentation, highlighting the novelty of our approach.

DL models are almost exclusively trained either from scratch or using existing large-scale datasets like ImageNet, with limited studies focusing on alternative pre-training approaches [3, 32, 33]. Training from scratch yields models heavily dependent on specific data, which can reduce their generalization performance on external datasets. Addressing this requires collecting extensive, costly radiological images and labels [34]. While cost-effective, ImageNet contains images (e.g., cats, birds) that are vastly different from medical images, potentially causing negative transfer and limiting performance. Nishio et al. addressed this by generating a lung cancer segmentation dataset using GANs and 3D graph cuts, eliminating the need for additional labeling [35]. This method is compelling as it is task-specific for lung cancer segmentation and incurs no additional labeling cost. However, GANs can overfit with limited data, producing noisy or low-variation (mode collapse) output [36].

For both the DeepLabV3+ and DPT models, FractalDB-1K_PT yielded better accuracy than training from scratch or ImageNet-1K_PT. Our subgroup analysis showed FractalDB-1K_PT led to greater improvements in accuracy than ImageNet-1K_PT, particularly in complex and irregular cases, as opposed to cases closer to a sphere. We posit this improvement in segmentation accuracy stems from pre-learning fractal structures, which constitute the fundamental shapes of lung cancer lesions.

Our best performing model (DPT + FractalDB-10K_PT), trained with 104 CT images, achieved a $\text{vDSC}$ of 0.800 ± 0.079. While a direct comparison is difficult due to differences in disease stages and models used, it is a notable result that our proposed model, fine-tuned with a relatively small amount of clinical data, achieved a comparable $\text{vDSC}$ to the average of 0.78 reported in previous studies [3]. Most prior studies utilized more clinical data or other modalities (e.g., PET/CT images) than our research, but such extensive data may not be collectible at all institutions [3, 32]. Misera et al. proposed a scalable AI that weakly depends on training data by combining pre-training with a small amount of clinical data, aiming to reduce data collection costs and enable rapid model construction [34]. Fractal pre-training aligns with their view, offering a scalable AI solution capable of achieving sufficient accuracy even with limited clinical data. This effectiveness stems from its dual advantages: (1) it eliminates the need for manual annotation by using generative parameters as pseudo-labels, and (2) it provides a morphologically relevant starting point for fine-tuning, enhancing data efficiency. Furthermore, FractalDB has been publicly released by Kataoka et al., with image generation code and pre-trained models readily available. This makes it a valuable resource for model construction using institution-specific data at any facility.

In our experiments, the ViT-based model, DPT, outperformed the CNN-based model, DeepLabV3+, in terms of $\text{vDSC}$ and $\text{sDSC}$ accuracy. Although a direct comparison is difficult due to the differences in training methods, this performance difference can be attributed to the architectural differences between the two models. Specifically, whereas CNN excels at extracting local features through convolutional operations, ViT can capture global features across the entire image using its self-attention mechanism. In medical image segmentation, where understanding the overall structure of objects and their spatial relationships is crucial, this capability of the ViT likely conferred a distinct advantage, leading to improved accuracy [37, 38].

**Fig.8:**
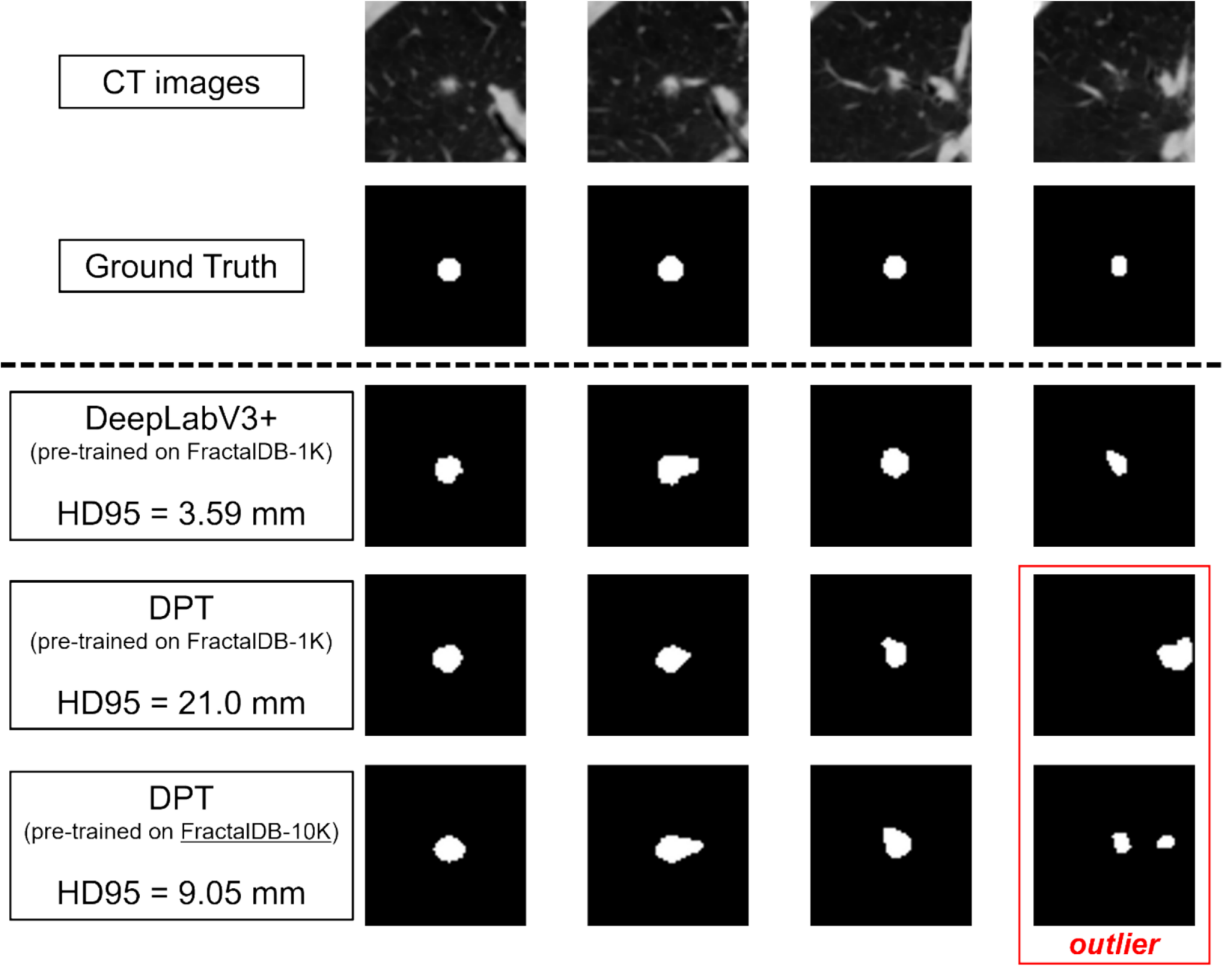
A representative example of a case with a poor $\text{HD95}$ score. The DPT model pre-trained on FractalDB-1K exhibits a large prediction error on a single slice. In contrast, the model pre-trained on FractalDB-10K shows a clear improvement, although a non-negligible error still persists. $\text{HD}95$, 95th percentile Hausdorff Distance

On the other hand, in terms of the $\text{HD95}$ score, DeepLabV3+ demonstrated higher accuracy, except when DPT was pre-trained on FractalDB-10K_PT. As shown in the box plot in Fig. 4, DPT exhibits a tendency for more outliers compared to DeepLabV3+. Indeed, in the case with the worst $\text{HD95}$ score for DPT (pre-trained on FractalDB-1K_PT), as illustrated in Fig. 8, the prediction for only a single slice deviated significantly from the ground truth. Such sporadic prediction failures are a known instability in ViT models, often caused by insufficient pre-training. ViT-based models require pre-training on a much larger dataset compared to CNNs to stabilize their performance, suggesting that the outliers observed in our experiment may be due to the inadequate scale of the pre-training dataset [39]. In fact, when pre-trained on the larger FractalDB-10K_PT dataset, the number of outliers in DPT's predictions decreased, and its average $\text{HD95}$ accuracy surpassed that of DeepLabV3+. As illustrated in Fig. 8, although some mispredictions remain with the FractalDB-10K_PT model, a clear improvement is visible. This result strongly suggests that pre-training on a 1K-level dataset was insufficient for our experimental setup and that a larger-scale dataset is necessary to stably unlock the full potential of ViT.

The results of this study raise the question regarding the optimal size of the pre-training dataset for early-stage lung cancer segmentation. In our experiment, accuracy consistently improved as the pre-training dataset was scaled up from Scratch (no pre-training) to FractalDB-1K_PT and FractalDB-10K_PT, and a performance plateau was not observed. While larger fractal datasets could theoretically improve performance, this approach faces two major constraints: the impractical computational cost of pre-training (e.g., FractalDB-300K_PT required special computational resources such as supercomputers or Tensor Processing Units [40]) and the inevitable point of diminishing returns. Given these limitations, we argue that the most balanced and practical approach is the one adopted in our study: fine-tuning large, publicly available models with smaller, local datasets. While the adoption of a model pre-trained on FractalDB-10K does not necessarily guarantee optimal performance, it was considered the most reasonable approach among the practical options, as FractalDB-10K was the largest FractalDB available at the time of our research. Nevertheless, a crucial future research endeavor is to build, evaluate, and publicly release an even larger pre-trained model, such as FractalDB-20K_PT, for anyone to use. While this presents significant challenges in terms of computational resources, it is a vital step for the continued advancement of the field.

This study has several limitations. First, while this study focused on lung cancer, similar fractal properties are reported in other malignancies, such as breast and hepatocellular carcinoma [41–43]. Future work should assess the generalizability of fractal pre-training across different cancer types. Second, our analysis was conducted in a 2D framework to allow for direct comparison with standard 2D datasets like ImageNet. Given that clinical data is inherently three-dimensional, extending our methodology to 3D segmentation using 3D fractal images is a crucial next step to potentially enhance performance [44]. Third, the ground-truth contours were delineated by several radiation oncologists, each with more than 7 years of experience. Although these contours have been reviewed by at least three experienced radiation oncologists, it is important to note that inter-observer variability may still exist. Fourth, as this study was conducted using data from a single institution, the generalizability of certain settings—such as the ROI cropping size and resolution, subgroup analysis results, and model training hyperparameters—may be limited.

In summary, pre-training with fractal images, inspired by lung cancer morphology, achieved higher segmentation accuracy compared to existing methods such as training from scratch or ImageNet pre-training.
